# Faculty perceptions of student plagiarism and interventions to tackle it: a multiphase mixed-methods study in Qatar

**DOI:** 10.1186/s12909-020-02205-2

**Published:** 2020-09-21

**Authors:** Mai A. Mahmoud, Ziyad R. Mahfoud, Ming-Jung Ho, John Shatzer

**Affiliations:** 1 Department of Medicine, Weill Cornell Medicine, 1320 York Avenue, HT-621, New York, NY 10021 USA; 2 Department of Medical Education, Weill Cornell Medicine, Doha, Qatar; 3 Department of Population Health Sciences, Weill Cornell Medicine, 1300 York Avenue, New York, NY 10065 USA; 4 Department of Family Medicine, Center for Innovation and Leadership in Education (CENTILE), Georgetown University Medical Center, MedStar Health, Washington, DC 20057 USA; 5 Johns Hopkins University School of Education, Baltimore, MD 21218 USA

**Keywords:** Medical students, Plagiarism intervention, International learners, Academic integrity

## Abstract

**Background:**

The widespread use of the internet and other digital resources has contributed to the escalation of plagiarism among medical students and students of other healthcare professions. Concerns were raised by faculty at Weill Cornell Medicine-Qatar (WCM-Q), a branch of Weill Cornell Medicine of Cornell University in New York, who had been observing plagiarism in students’ assignments.

**Methods:**

To identify the extent of plagiarism practices and their contributing factors, a two-phase mixed-method research study was conducted, comprising a survey administered in 2013, followed by longitudinal interventions, and a second survey in 2017 to measure the impact of the interventions.

**Results:**

By Phase II, overall observed plagiarism incidents per year decreased from 44 to 28%, and the number of faculty who observed no plagiarism incidents increased significantly from 12 to 37%. The faculty concerned about student plagiarism decreased by 33% [53.1 to 20%] between Phase I and Phase II.

**Conclusion:**

When students are provided with information regarding what constitutes plagiarism and their institution’s policy in response to plagiarism incidents, they are less likely to engage in such practices.

## Background

### Plagiarism among students in healthcare professions

The definition of plagiarism, according to Merriam-Webster’s Learner’s Dictionary, is “the act of using the words or ideas of another person without giving credit to that person” [1]. Plagiarism has become a global problem in healthcare education, [2–5] escalated by the widespread use of the internet and other digital resources [2–4]. Donald McCabe, founder of the International Center for Academic Integrity, recognized these findings in his study of academic dishonesty within nursing schools, which found that the use of readily available electronic resources contributed to the growing incidence of plagiarism among nursing students [5]. Another study surveying students and faculty at 62 dental schools in the US and Canada revealed serious violations of academic integrity (AI) and plagiarism in course assignments and cheating in exams [6]. Students often cited common practice among peers and a lack of serious consequences or policy implementation, as well as stress, time pressure, and higher expectations from faculty among the reasons for widespread plagiarism and other forms of academic dishonesty [5].

Outside North America, Nilston et al. studied scientific dishonesty (SD) among postgraduates students (PhD) in Sweden and found that less than one third of the responders declared knowledge of the principles of research ethics like plagiarism or falsifying data [7]. The absence of a complete and comprehensive written policy on academic misconduct as reported in the Swedish study [7] is congruent with similar studies conducted in the US and Canada [5, 6].

Relatively few studies have been published about AI in the Middle East. In one study by Elzubeir et al. on educational dishonesty in the United Arab Emirates (UAE), 93% of senior medical students and interns surveyed considered academic misconduct, such as misuse of power, a serious violation of AI. However, only 38% of respondents considered copying and pasting from textbooks or citing published work without proper citation wrong [8]. A more recent survey in the UAE by Abdulrahman et al. on academic dishonesty revealed that 12% of students copied work from the record books (log books where students report their assigned tasks) of others, and 86% considered the act unethical [9]. While the research suggested instructing students in moral principles, no clear intervention was suggested to prevent the unethical behaviors [9].

In Saudi Arabia, a few studies conducted by Babelli et al. [10] and Sattar et al. [11, 12] on professionalism using the Dundee Polyprofessionalism Inventory^.^ concluded that self-plagiarism was not a major concern from the students’ point of view [10–12]. However, the paper does not clarify whether students received any formal teaching about plagiarism, or whether plagiarism in that setting had a social or cultural interpretation different from other countries.

### Plagiarism at Weill Cornell Medicine-Qatar

Established in 2002, Weill Cornell Medicine-Qatar (WCM-Q) is a branch of Weill Cornell Medicine of Cornell University in New York (WCM-NY) and the first medical school in Qatar. In implementing a North American medical education model, one challenge confronting WCM-Q is cultural in nature: WCM-Q students join the two-year premedical program directly from high school. They mostly hail from the region and are younger (the average age is 19 years on admission) than their peers in North America. For most, English is their second language and, perhaps more importantly, the extent of their knowledge of plagiarism prior to joining WCM-Q is often unclear. In informal meetings, faculty noted that some students seemed to be hearing the word “plagiarism” for the first time when brought up regarding submitted work. In the faculty’s view, the students were not aware of what constitutes plagiarism or its negative consequences on one’s professional career. The faculty also noted that in comparison to the US, the concept of plagiarism and its ethical values are not explicitly taught in the school system in Qatar or in the region. Moreover, in most cases, our students transition directly from high school to WCM-Q’s rigorous premedical courses, and consequently this—along with their overall younger age—likely impact their ability to handle stress and time management, thus contributing to plagiarism at our school.

To our knowledge, ours is the first study in the State of Qatar and in the Middle East region to examine the impact of structured educational intervention to tackle plagiarism. As a North American institution in the Middle East serving multicultural students with diverse educational backgrounds, we believe that our study can contribute to the global medical education community, especially in light of rising interest from other institutions in replicating our model [13].

## Methods

To address faculty concerns about plagiarism at WCM-Q and better understand student behaviors, we sought to identify the extent of plagiarism practices and their contributing factors through purposeful sampling. To this end, we designed a convergent parallel mixed-method study to collect qualitative and quantitative data in a two-phase approach for separate analysis [14]. Phase I consisted of a survey administered to faculty members in 2013, followed by anti-plagiarism interventions in teaching, learning, and policy based on the Phase I survey results. Phase II consisted of a second modified survey distributed in 2017 to assess the effect of the interventions.

### Instruments

The surveys were developed by faculty with expertise in survey design and knowledge on the topic of plagiarism, and were piloted and compared to similar surveys published in the literature [15, 16]. Both contained a qualitative and a quantitative section. In the qualitative section, open-ended questions asked participants to identify factors that contribute to student plagiarism, propose an effective anti-plagiarism intervention, and describe how they respond to plagiarism and its consequences. The quantitative section included rated scales to gather information on demographics, teaching experience in general, the frequency with which plagiarism was encountered, and awareness of the school plagiarism policy.

Both surveys included statements on the study’s research objectives, data security, and consent to participate in the research (Additional file 1). Ethics approval was obtained from WCM-Q’s Institution Review Board (IRB 0003) (Phase I and II) and Johns Hopkins University IRB HIRB00005861 (Phase II).

### Phase I (2013)

#### Data collection

Anonymous paper surveys were distributed in sealed envelopes by division secretaries to all 74 premedical and medical faculty members. Due to the integrated six-year premedical and medical program, as well as the small number of faculty teaching exclusively in either track, the findings of these two faculty groups were not separated. The completed surveys were returned with no identifiers in a visibly located and closed box.

### Interventions (2014–2017)

In Phase I, when faculty were asked to recommend an intervention they felt would be effective in decreasing plagiarism on assignments, most suggested education about plagiarism and its ethical value, use of software to check for plagiarism, and a clear policy. These results prompted the following longitudinal interventions:

#### Plagiarism policy and dissemination

A clearly articulated statement about plagiarism was added to all courses and clerkships syllabi, and explicitly discussed in the orientation sessions for those courses. The policy was also published in the WCM-Q student handbook, and first-year students signed a pledge on plagiarism and AI during their orientation week.

#### Online tutorials and plagiarism seminars

Seminars and *t*utorials were implemented with the overall objective to address missing knowledge on plagiarism. Ninety-minute plagiarism seminars were delivered longitudinally every year for pre-clinical students by both faculty and librarians and included examples of plagiarism and its consequences. A locally developed, mandatory online tutorial with pre- and post-tests was assigned to pre-clinical students before they started their clerkships to remind them of the concept and consequences of plagiarism.

#### Plagiarism detection software

Turnitin® anti-plagiarism software was embedded in course websites and implemented for all assignments. When students uploaded their work, Turnitin immediately reported a similarity score; a high similarity score (> 10%) warned students and faculty to check for plagiarism. This score helped students and faculty intervene accordingly.

#### Student resources

Students were encouraged to reach out to WCM-Q’s clinical psychologist and two academic counselors to help handle stress and gain skills in time management. While the student academic counseling service had been established before Phase I, an in-house psychologist was hired in 2015 by the Department of Student Affairs to make the service more accessible and maintain student confidentiality.

### Phase II (2017)

#### Data collection

Repeating the approach in Phase I, surveys were distributed to all 69 premedical and medical faculty members. The Phase II surveys included questions from Phase I as well as four new questions that aimed to measure the impact of the anti-plagiarism interventions that were put in place as a result of the Phase I responses. The new questions asked whether a participant had participated in the Phase I survey, their perception of any changes in plagiarism incidence over the last 4 years, and whether s/he had implemented a new plagiarism strategy, and if so, to identify it from the list.

### Phase I and phase II: data analysis

Descriptive analyses were conducted for both quantitative and qualitative data and statistical analysis was performed for the quantitative section. Faculty member perceptions of the extent of plagiarism and their awareness of the institutional policy were compared across Phase I and Phase II, and the impact of the interventions was assessed and analyzed. Although the majority of participants may have answered both surveys, no indicators linked their answers. Triangulation of the qualitative data was used to explain and interpret the quantitative data.

#### Statistical analysis

Frequency distributions (counts and percentages) were used to summarize all demographic and plagiarism related questions. These were performed separately for the cohort of survey participants in Phase I and in Phase II. Changes in responses between the phases were assessed using the Chi-square test (or Fisher’s exact test when expected cell counts fell below five). For the numeric variable, or number of incidents of plagiarism encountered, the Wilcoxon Rank Sum test was used to check for differences between the two phases.

All analyses were conducted using IBM-SPSS (version 23). A *p*-value of 0.05 or less was considered statistically significant.

#### Qualitative analysis

Answers to the open-ended questions were analyzed by two authors using a qualitative thematic analysis approach following the constructivism research philosophy. Two researchers identified themes that emerged from the qualitative data then compared the themes. They discussed each point of disagreement until reaching consensus.

## Results

Thirty-five faculty members (47.2% response rate) in Phase I and 33 faculty members (47.8% response rate) in Phase II completed the survey. As shown in Table 1, there were no statistical differences between the two cohorts in gender, place of the most educational experience, and teaching experience. In Phase I, 34.3% were female; in Phase II, 36.4% were female. About 40 to 60% of the respondents (Phase I and II respectively) had more than 10 years of experience teaching, and a significant percentage (44%) had been employed for more than 10 years at WCM-Q. The majority gained most of their teaching experience in North America.

**Table 1:** Demographic and work characteristics of faculty participants in both phases

		Phase I (***N*** = 35)	Phase II (***N*** = 33)	
		N(%)	N(%)	***P***-value
**Gender**	Female	12 (34.3%)	12 (36.4%)	0.085
	Male	23 (65.7%)	21 (63.6%)	
**Educational level**	Undergraduate/BA/BS	7 (20.0%)	0 (0.0%)	0.015a
	Graduate (Masters)	2 (5.7%)	2 (6.1%)	
	PhD	13 (37.1%)	13 (39.4%)	
	MD	11 (31.4%)	18 (54.5%)	
	Other	2 (5.7%)	0 (0.0%)	
**Where do you teach?**	Premed Program	15 (42.9%)	9 (27.3%)	0.041^a^
	Medical Program	18 (51.4%)	22 (66.7%)	
	Both	2 (5.7%)	2 (6.1%)	
**Where did you have most of your teaching experience?**	GCC^b^	9 (25.7%)	3 (9.4%)	0.149
	North America	18 (51.4%)	14 (43.8%)	
	Europe	3 (8.6%)	4 (12.5%)	
	Asia	1 (2.9%)	4 (12.5%)	
	Multiple	4 (11.4%)	4 (12.5%)	
	Others	0 (0.0%)	3 (9.4%)	
**Years of teaching medical/premedical students**	0–10 years	20 (57.1%)	13 (39.4%)	0.143
	> 11 years	15 (42.9%)	20 (60.6%)	

^a^ Significant difference between the two phases at the 5% level

^b^ GCC is the Gulf Cooperation Council: Qatar, Bahrain, Kuwait, Oman, Saudi Arabia, and United Arab Emirates

When asked about plagiarism incidents per year, 37.5% of faculty in Phase II reported no cases encountered, compared to 12.1% in Phase I (*p* = 0.005, Table 2): a statistically significant decrease in the second phase.

**Table 2:** Frequency of plagiarized work in phases I and II, and faculty responses to plagiarized work

Faculty responses to surveys		Phase I N (%)	Phase II N (%)	***P***-value
Overall frequency of plagiarism identified by faculty	None	4 (12.1%)	12 (37.5%)	0.005^a^
	>Once/year	14 (42.4%)	9 (28.1%)	0.425
	>Once/month	12 (36.4%)	4 (12.5%)	0.051
Faculty concerned about reporting student plagiarism?	Yes	17 (53.1%)	6 (20.0%)	0.007 ^a^
	No	15 (46.9%)	24 (80.0%)	
**Faculty responses to student plagiarism**				
● Do nothing		2/29^b^ (6.9%)	1/24^b^ (4.2%)	0.999
● Communicate with student		28/29 (96.6%)	22/24 (91.7%)	0.584
● Report to offices within institution		6/29 (20.7%)	5/24 (20.8%)	0.990

^a^ Significant difference between the two phases at the 5% level

^b^ Among those who answered this question. Note that faculty members can choose more than one answer

We also found that the concerns about reporting student plagiarism dropped from 53.1 to 20% (*p* = 0.007, Table 2). As shown in Table 2, the majority of faculty members preferred to address the plagiarized work with students individually, rather than reporting them to school officials.

The results from the questions on faculty awareness of the institution’s policy for plagiarism revealed no statistical differences in faculty knowledge of such a policy or where they could find a copy of the policy.

Our qualitative analysis identified eight themes from the answers to the open-ended questions on factors contributing to student plagiarism. Of those themes, ignorance or lack of conceptual knowledge about plagiarism ranked highest across cohorts: more than half of the comments in Phase I and more than one-third in Phase II. Time management ranked second in Phase I and II respectively. Laziness and lack of consequences ranked higher in 2013 than in 2017. The common themes are shown in Table 3 with representative quotes.

**Table 3:** Thematic analysis of faculty perceptions of factors contributing to student plagiarism

Themes	Representative quotes (2013)	Representative quotes (2017)
Lack of plagiarism concept	“Value place on plagiarism seems to be different here from North America”	“Lack of awareness of what constitutes plagiarism”
	“Despite education, not clear on what plagiarism is”	“Lack of clarity of plagiarism vs quotation”
	“Unaware of what constitutes plagiarism and what constitutes working together”	“Not understanding that minimal paraphrasing of a sentence does not mean this is not a copy”
	“They believe they are helping each other”	
	“Not knowing this behavior is not allowed”	“Unsure of why they should not plagiarize”
	“Sometimes unintentional”	
	“Lack of knowledge” “ignorance”	
Time management	“Time pressure”	“Time restrictions”
	“To save time”	“Time constrain with heavy schedule”
	“They do not have time”	“Pressure to have the work done on time”
	“Time management”	
Laziness	“Thought of presenting a good write up without committing the necessary effort”	“Laziness”
	“Laziness is number 1 in areas I discover. However, in first year, it can be a lack of understanding expectations but this is corrected very early.”	“Laziness or being unsure of the importance of doing their own work”
		“Easy copy and pasting”
	“Laziness; lack of motivation”	
	“Leaving assignments till last minutes and rushing to finish”	
Lack of consequences	“Have been no serious consequences here that I know of”	“No clear consequences”
	“They feel they won’t get caught”	
	“Everyone else does it”	
Pressure to do well	“Pressure to achieve high marks”	“Pressure to success”
Academic difficulty		“Difficulty with subject matter”
		“Lack of self-confidence, poor sense of values”
Language weaknesses	“Language difficulty”	“Language difficulty”
Carelessness	“Carelessness”	“Carelessness”
	“Poor use of resources”	

Faculty were asked to nominate up to three interventions that in their opinion would help (Phase I) or had helped (Phase II) students to avoid plagiarism. Education about plagiarism (seminars and tutorials), clear policy, and the use of the software in assignments ranked highest among effective interventions in Phase II (Table 4).

**Table 4:** Faculty perceptions of effective interventions for tackling plagiarism (data from phase II survey, 2017)

Anti-plagiarism intervention	Percentage chosen
Clearly written policy	27
Education about plagiarism	25
Software (Turnitin®)	24
Statement in the syllabi	10
Statement in the student handbook	8
Not sure	6

## Discussion

Reasons for plagiarism most likely are multifactorial, and a combination of these factors can cause a student to plagiarize work either once or repeatedly. Our faculty members ranked lack of knowledge about plagiarism or insufficient understanding of the ethics associated with it as a primary cause. This finding is also consistent with studies from Europe and the Middle East [7, 8], as well as others comparing students from the Middle East to their counterparts in the UK that suggest cultural background as a factor [10, 12, 17]. Our results are also partially congruent with those of Heitman and Litewka [18] in their study of plagiarism among international graduates seeking postgraduate training or education in the US. The authors posit that lack of knowledge regarding what constitutes plagiarism, along with difficulty with the English language, were the main reasons for students plagiarizing work.

Although most of our students do not claim English as their native language, WCM-Q requires English proficiency for acceptance to its program. Moreover, the first 2 years of its curriculum include two first-year writing seminars conducted in English and assessed according to Cornell University standards. While evaluation of these seminars was not part of our study, the rigor of the seminars supports why language difficulties were not a major concern.

However, the faculty observation that their students believed paraphrasing and self-plagiarism entailed less serious acts of academic dishonesty does suggest a lack of knowledge about it; this is supported by the reduced incidents of plagiarism in Phase II, which indicate that the students grew more aware of plagiarism as they advanced in their learning. In comparison, evidence from the UAE study showed no difference in plagiarism (work and ideas) as students advanced in their medical school [9].

WCM-Q faculty members also pointed out a lack of clear policy, among other concerns, and noted a preference for addressing incidents of plagiarism with students directly. In studies by Babelli and Guraya comparing faculty in the UK and Saudi Arabia, both groups recommended failing the student from the course as a sanction for some types of plagiarism such as paraphrasing [10, 17]. Our faculty are well aware of the ethical and professional aspects of plagiarism and its consequences; however, as they believe lack of knowledge is the major contributor, they prefer to educate students about plagiarism first and then assess for correction or a pattern of the behavior.

We also agree with the recommendations of Heitman and Litewka [18] to change the perception of plagiarism as normal. To address this normalization, we implemented comprehensive interventions (illustrated in Table 4) that encompassed active learning and deliberate practice. We applied principles of adult learning theory by choosing relevant and diverse instructional methods to reinforce the concepts of plagiarism and its consequences [19]. For example, a policy for plagiarism and AI was put in place and made visible to both faculty and students. We emphasized cultural ethical values by translating “plagiarism” across languages so students could recognize its meaning in their native tongue. A systematic review by Guraya and Guraya [20] also proposed some of these interventions, such as raising awareness of the legal aspects of plagiarism and introducing an approach in academic institutions, which were relevant to our context as a multicultural school.

While more premedical faculty participated in Phase I (42.9%) than in Phase II (27.3%), which may suggest a natural development in plagiarism awareness independent of our study, the faculty comments in Phase II (Table 2) and the anti-plagiarism interventions they had identified as effective contradict this assumption. Furthermore, students are unlikely to change their behaviors without intervention. The response rate may have been impacted by non-response bias to a sensitive issue, potentially influencing the estimation of plagiarism incidence in the sample. The comparable response rate in both study phases and the use of close-ended questions in the quantitative portion decrease that bias, however. The consistency of similar responses to plagiarism factors and the comments (quoted in Table 3) added to the dependability of our study.

Our results show that most of our interventions were effective in raising alertness among students. Although faculty responses to student plagiarism did not change significantly over the two phases of the study, awareness of the policy contributed to a significant drop in students plagiarizing in Phase II. However, more work has to be done, at least for faculty, to increase awareness of the AI policy and consistency in responding to student violations of it.

### Limitations

Due to the small sample size in both phases, the study might not be powered to detect considerable and important trends that could reflect possible changes in aspects of plagiarism at WCM-Q. Additionally, the survey response rate of 47.2 and 47.8% may not represent all faculty at the university, and hence results might not be generalizable.

Finally, the internal validity of the study could have been affected by possible confounders, such as the type of students and courses that faculty members were teaching, and any changes in student awareness about plagiarism that had happened during the 4 years of this study.

## Conclusion

This is the first study of its kind to look at trends at WCM-Q. The results of this study could create a baseline for future research and be used to estimate proper sample sizes for future trend studies. The diverse group of participants allows for a potential general overview of the situation and how it changes over time. Deliberate and focused teaching on plagiarism should be the practice in all colleges in the region and globally for those who take international students. Differences in cultural understanding about the definition and ethics of plagiarism can easily be corrected through implementing our anti-plagiarism interventions, which successfully improved our students’ knowledge of and attitude toward plagiarism.

## Supplementary information

**Additional file 1.**

## Data Availability

The data sets used and analyzed during the current study are available from the corresponding author on reasonable request.

## Abbreviations

WCM-Q: Weill Cornell Medicine-Qatar
WCM-NY: Weill Cornell Medicine of Cornell University in New York
AI: Academic Integrity
IRB: Institutional Review Board
